# Revisiting non‐obstructive azoospermia: Is there a best way to retrieve testicular sperm?

**DOI:** 10.1002/rmb2.12632

**Published:** 2025-02-06

**Authors:** Satoru Kanto, Kentaro Ichioka, Yuri Sato, Yoshihiko Uchino, Takayuki Tanaka, Mareyuki Endo

**Affiliations:** ^1^ The Kanto Clinic Sendai Japan; ^2^ Men's Fertility Clinic Tokyo Tokyo Japan; ^3^ Osaka General Hospital of West Osaka Japan; ^4^ Torch Clinic Shibuya Japan; ^5^ Sendai Kousei Hospital Sendai Japan

**Keywords:** azoospermia, FNA mapping, microdissection TESE, non‐obstructive azoospermia, TESA

## Abstract

**Background:**

Microdissection TESE has been considered the “gold standard” for retrieving testicular sperm in cases of non‐obstructive azoospermia (NOA) despite limited scientific support. Here we compare all aspects of microdissection TESE with testis fine needle aspiration mapping (FNA Mapping) and directed TESE procedures for men with NOA.

**Methods:**

We examine the history of testicular sperm extraction techniques and the rise of advanced technologies with a focus on microdissection TESE and FNA mapping. We summarize the published literature regarding the success rates, complications, and limitations of these two methods.

**Main Findings:**

As there are no randomized controlled trials, the best data come from the Cochrane Reviews, which include meta‐analyses concluding that the simplest and safest methods of sperm retrieval should be chosen. Although microdissection TESE is popular, recent reports have questioned its value due to the significant hypogonadal consequences. Among alternative procedures, FNA Mapping is a viable and less invasive alternative to microdissection TESE in finding testicular sperm in NOA patients.

**Conclusion:**

Alternatives to microdissection TESE procedures such as FNA Mapping offer several advantages that include similar sperm retrieval success rates, but also less invasiveness and improved understanding of the pathophysiology of NOA.

## 1 INTRODUCTION

Intracytoplasmic sperm injection has made it possible to offer biological fatherhood to even azoospermic couples.^1^ Azoospermia is categorized into obstructive (OA) and non‐obstructive (NOA) forms on the basis of past medical history, testicular volume, follicle‐stimulating hormone (FSH) level, and genetic findings including chromosome testing, and Y chromosome azoospermic factor (AZF) deletion, with the final diagnosis historically depending on the histopathology of testis biopsy procedures.^2^ In azoospermic men, the retrieval of testicular sperm has been the primary concern for clinicians, leading to the obsolescence of diagnostic testicular biopsy and seminal vasography^3^, ^4^ to make the diagnosis. However, the preoperative diagnosis of OA and NOA is often subjective based on these clinical findings, which can sometimes result in inappropriate surgical procedures and failure to retrieve testicular sperm. More recently, NOA can be assessed by sperm findings during testicular sperm retrieval procedures in which few or no sperm are found.

**FIGURE 1:**
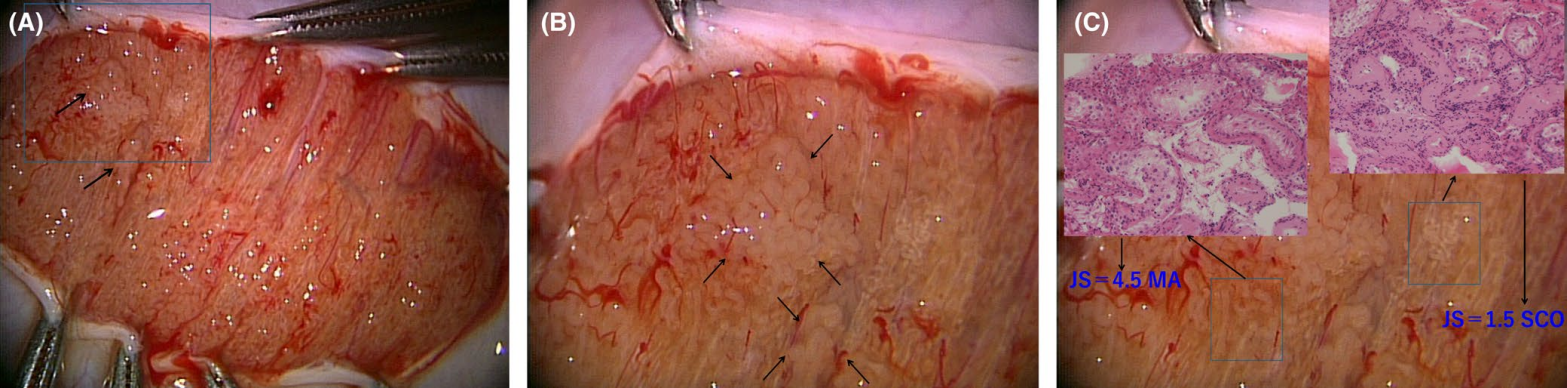
Histopathology depends on the biopsy extraction site in NOA. (A) Testicular sperm was retrieved from area indicated by the arrows. (B) Heterogeneity of seminiferous tubules was easily observed. (C) Discrepancy of pathological diagnosis depends on extraction site.

With diagnostic testicular biopsy, the pathology explaining NOA has been categorized into three types: Sertoli Cell Only (SCO),^5^, ^6^ Maturation Arrest (MA),^7^, ^8^ and Hypospermatogenesis (HS). However, NOA testicles commonly have “islands” or “patches” of sperm present in only focal areas of the testis.^9^, ^10^ This heterogeneity of spermatogenesis has taught us that a single or few tissue specimens do not necessarily represent the biology of the whole testis, especially if the specimen is picked up arbitrarily under microdissection TESE (Figure 1A–C). This variability in NOA testis histology makes it challenging to: (a) accurately predict whether a specific histological pattern will lead to a successful sperm retrieval and (b) evaluate and compare the results of the various sperm retrieval procedures used in NOA patients.

Many approaches have been taken to find sperm in NOA patients, including conventional TESE procedures, multibiopsy TESE procedures, microdissection TESE and FNA Mapping^11^, ^12^ followed by directed TESE procedures. Microdissection TESE is currently the most popular technique, and it has been adopted worldwide. However, there is a substantial literature to support the use of alternative approaches such as FNA Mapping and map‐directed TESE procedures as equally viable options for NOA patients. This review presents the currently published evidence that supports this statement.

## 2 MATERIALS AND METHODS

We searched for and compiled the existing medical literature on the PubMed database using the keywords “azoospermia”, “TESA”, “FNA mapping”, “microdissection”, “TESE”, “sperm retrieval”, and “hypogonadism”. The keyword “azoospermia” was a mandatory keyword for literature included in this study. The comprehensive literature search was performed from January 2024 to July 2024. We reviewed all studies and focused on those describing sperm retrieval techniques in NOA. We carefully checked each study for an objective definition of NOA that included reports of histopathological diagnoses. We also endeavored to find a rule for selecting tissues to use in pathological diagnosis with microdissection TESE. We thus conducted a narrative review while considering underlying questions about the definitions of NOA and conventional pathological diagnosis.

## 3 MAIN FINDINGS

### 3.1 Historical background of azoospermia diagnosis and sperm retrieval techniques

Prior to the era of TESE, azoospermia was diagnosed by testicular biopsy and seminal vasography. Cases of normal spermatogenesis and/or obstruction of the seminal pathway were diagnosed as OA. Cases of impaired spermatogenesis or aspermatogenesis were diagnosed as NOA and categorized into three pathological types: SCO,^5^, ^6^ MA,^7^, ^8^ and HS. The first report of ICSI was published in 1992,^1^ and the first report of TESE‐ICSI was published in 1995.^13^ Because TESE provides information on the type of azoospermia and can bypass infertility by providing sperm for IVF‐ICSI, diagnostic testicular biopsy and seminal vasography have been largely abandoned, and clinical interest has focused more on testicular sperm retrieval rather than understanding the etiology of azoospermia.

The heterogeneity of spermatogenesis in the testes was first demonstrated with FNA Mapping in 1997,^11^, ^12^ followed by microdissection TESE in 1998.^14^, ^15^ FNA Mapping met with early acceptance as an effective technique for locating sperm as it was able to find sperm when testis biopsies failed to do so.^11^ Microdissection TESE was also accepted as an effective sperm retrieval technique in NOA as it demonstrated a better yield of sperm when compared to conventional TESE techniques.^15^ The operating microscope detects focal sperm producing regions because, in principle, seminiferous tubules containing developing germ cells, rather than Sertoli cells alone, are likely to be larger and more opaque than those without sperm production.^16^ This procedure was recognized as the most effective and minimally invasive technique because operating under the aforementioned principle was believed to result in the selection of only promising seminiferous tubules during the first surgical procedure.^17^ Microdissection TESE has become the more popular technique for testicular sperm retrieval despite there being no rigorous research or clinical trials showing its superiority to testis FNA mapping.^16^, ^17^, ^18^

To its credit, for non‐mosaic Klinefelter syndrome, microdissection TESE is the most effective procedure for retrieving testicular sperm.^19^ Fertilization rates, pregnancy rates, and live birth rates have been excellent in KS patients using this technique.^20^, ^21^, ^22^ Initially, fresh microdissection TESE was performed simultaneously with oocyte retrieval.^23^ However, since the establishment of testicular sperm cryopreservation, microdissection TESE can now be performed first and sperm frozen, followed by oocyte retrieval at a later date.^24^, ^25^ Microdissection TESE has also been reported to be effective in patients with spinal cord injuries.^26^, ^27^ Lastly, the side effects of microdissection TESE were reported initially as minimal, with hypogonadism reported to be unlikely to occur after the procedure.^28^

In light of these considerations, microdissection TESE has become more widely accepted in many countries. In Japan, microdissection TESE has been covered by public health insurance since 2022. The Japanese Urological Association (JUA) published the *Clinical Practice Guideline for Male Infertility* in 2024, in which it gives a ‘grade A recommendation’ (strongly recommended) to microdissection TESE, despite evidence being limited to only level II: based on case control studies and repeated empirical observations.^29^ It is important to note that there have been no randomized controlled trials (RCTs) with microdissection TESE showing its superiority to any other sperm retrieval technique, including FNA mapping. Lastly, we could not find objective criteria distinguishing between OA and NOA, nor any guidelines for selecting tissues to pick up for assessment of histopathology during microdissection TESE, which significantly limits the diagnostic information learned from this technique.

### 3.2 Evidence against the superiority of microdissection TESE

Most physicians believe microdissection TESE is superior to every other testicular sperm retrieval technique, such as conventional TESE, TESA, or Open Testicular Mapping (OTEM).^30^, ^31^ Notably, in the absence of Randomized Controlled Trials (RCTs), the best data are derived from Cochrane Reviews, which performed a meta‐analysis on this topic and concluded that: (a) no particular sperm retrieval procedure is recommended over any other based on the lack of RCTs^32^, ^33^ and (b) that the simplest and safest methods should be chosen for sperm retrieval.^34^, ^35^

Recently, studies challenging the superiority of microdissection TESE have been published.^36^ Several crucial points are evident from this. First, false‐negative cases of microdissection TESE have been reported in as high as 29% of cases.^37^ Why do false‐negative cases occur with microdissection TESE? One reason authors propose that it is because microdissection TESE procedures tend to sample tissue centrally in the testis but not as well in the peripheral testis,^38^ which can limit its precision. Also since in microdissection TESE procedures, the heterogeneity of seminiferous tubule diameter is the most favorable predictor in retrieving testicular sperm,^39^ some posit that the visual nature of the technique is not precise enough to accurately differentiate sperm‐containing tubules from non‐sperm‐containing ones in all cases. In fact, the ability of microdissection TESE to find sperm in cases of MA is actually no better than a conventional TESE procedure, as sperm‐negative tubules affected by MA appear microsurgically virtually identical to normal, sperm‐producing ones.^38^

In addition, in some SCO cases, and most cases of MA, there are very subtle differences, or no differences at all, between the appearance of seminiferous tubules with spermatogenesis and those without spermatogenesis under operative microscopy. Correlating with this, it has been reported that the predominant histologic patterns present when sperm are detected by FNA Mapping after failed microdissection TESE procedures are the SCO and MA patterns.^37^

Second, it has also been reported that there is a significant rate of surgically‐induced hypogonadism after microdissection TESE procedures.^40^ This is because even with surgical microscopy, focal spermatogenesis cannot be recognized if it is located deeply inside the testes or is far from the incision line of the tunica albuginea. If focal spermatogenesis is not located near the surface, tissues must be dissected or divided to search deeper inside the testes. However, it is difficult to search the entire testes without causing damage because Leydig cells‐which produce testosterone and are located adjacent to the seminiferous tubules‐can be injured or removed when seminiferous tubules are dissected or divided. This can lead to injury or gross removal of Leydig cell populations adherent to the excised tubules. Even in cases where serum testosterone levels do not decrease after microdissection TESE, increased levels of luteinizing hormone are usually observed,^40^ which reflects the decrease in Leydig cell function.

When microdissection TESE was first reported by several pioneer surgeons, serum testosterone levels did not appear to decrease permanently post‐procedure, except in cases of Klinefelter syndrome.^41^ Currently, however, with a longer and broader history of this technique, the occurrence of hypogonadism as an aftereffect appears substantially more commonly,^42^ suggesting it might have been underestimated. Recently, sustained decrease in testosterone level was reported to occur after failed microdissection TESE in 10–35% of cases.^40^, ^42^ In contrast, there are no published reports of either testicular scarring changes in testis size or hypogonadism after FNA mapping since its original description in 1997.^11^

### 3.3 Microdissection TESE is not needed in Most NOA cases

**FIGURE 2:**
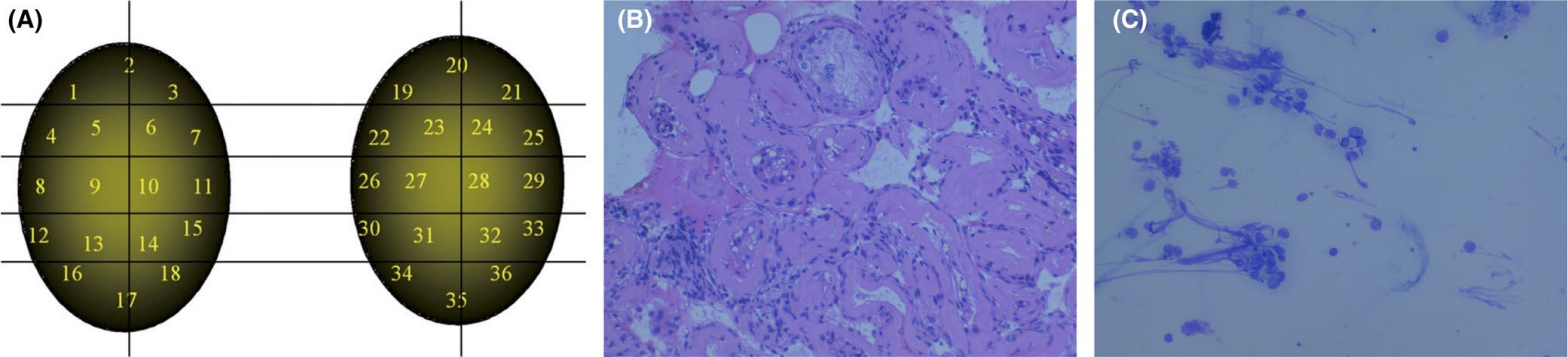
TESA was enough to retrieve testicular sperm for ICSI even in SCO. TESA succeeded in freezing testicular sperm into 10 vials. With thawing one of them on the day of retrieving oocytes, the partner in this case became pregnant after the first ICSI cycle. (A) Standard FNA Map sample size. It is divided into 18 sites/testis and performed in a grid‐like manner. (B) FNA testicular biopsy demonstrating SCO histology pattern. (C) An FNA stained cytology slide from a single testicular site showing sperm.

Microdissection TESE is quite effective in detecting focal spermatogenesis especially in cases of SCO histology^43^ (Figure 1A–C). However, simpler conventional TESE or TESA procedures used most commonly for sperm retrieval in OA cases have been shown to be effective in retrieving testicular sperm in SCO cases after detecting the location of sperm pockets with FNA Mapping.^44^ This raises an important question: “Is microdissection TESE necessary to retrieve testicular sperm in all NOA cases?” This may not be true in cases where focal spermatogenesis is detected in multiple anatomical sites by FNA Mapping.^45^ In these instances, TESA succeeds in retrieving enough testicular sperm for ICSI, making microdissection TESE unnecessary (Figure 2A–C).

In fact, in about half of NOA cases, TESA or conventional TESE is sufficient to retrieve testicular sperm after FNA Mapping (Table 1). Even in SCO cases, TESA can retrieve enough testicular sperm if multiple sites of sperm are found on FNA Mapping. For extremely challenging SCO cases, FNA Mapping combined with targeted microdissection TESE is an incredibly effective approach, procuring sufficient sperm to inseminate all eggs during IVF in 92% of cases.^46^

**TABLE 1:** Sperm retrieval outcomes after FNA Mapping in consecutive NOA patients: (*n* = 223).

Sperm retrieval procedure	Patients with sperm on FNA mapping	% of cases	Successful sperm retrieval (%)
TESA	46	43%	100%
cTESE	35	33%	100%
microTESE	24	23%	92%

*Note*: Ref: Turek PJ. Non‐Microsurgical Testis Sperm Extraction. In: Encyclopedia of Reproduction. 3rd edition. Ed: Michael Skinner, Academic Press, 2024.

In summary, early on after it was described, there was a strong belief that microdissection TESE was a less invasive and more effective procedure than either TESE or TESA techniques in all cases of NOA. However, more recent studies indicate that bilateral microdissection TESE is not necessarily less invasive,^47^, ^48^ may not be a technically superior technique, and may cause hypogonadism in a significant proportion of cases. Thus, from a procedural point of view, FNA Mapping should be considered an equally viable alternative sperm detection technique to microdissection TESE. And certainly, long‐term endocrinological follow‐up is necessary after microdissection TESE to better understand its long‐term effects and determine its true level of invasiveness.^49^

### 3.4 A comparison of current approaches to defining testis pathology in NOA cases

Azoospermia has been classified as either OA or NOA.^2^ While diagnostic testicular biopsies were used in the past, the final histological diagnosis of NOA is now made during TESE or microdissection TESE procedures. However, given the heterogeneity of spermatogenesis in the testes, neither approach, especially when tissue is collected arbitrarily under surgical microscopy, necessarily represents the entire pathology of the whole organ. The conventional classification of testicular pathology assumed that a randomly picked biopsy specimen represents the entire testis and that spermatogenesis level is homogenous throughout the testis. But as we now know from microdissection TESE and FNA Mapping procedures that this is not true. Thus, the conventional classification of pathology really only describes the histological pattern of a local, biopsied area and not the overall pattern found throughout the testis. This logistical problem in testicular pathology should be further considered to correctly address the reporting inaccuracies that challenge us in cases of NOA azoospermia.

Previous research indicates that cytology obtained by FNA is highly correlated with histopathology obtained by simple open biopsy.^50^, ^51^, ^52^, ^53^, ^54^, ^55^ However, different diagnostic skills are needed in cytology than in pathology because cytology is evaluated independently of tissue architecture.^56^, ^57^ A major advantage of FNA biopsy is that all germ line spermatogenic cells derived from a tissue specimen can be easily identified, indicating mature sperm with tails. Notably, it is often very difficult to see sperm tails on histopathology specimens due to cell crowding. As an example, FNA Mapping is far superior to testis histopathology in differentiating late maturation arrest at the spermatid stage from normal spermatogenesis. Thus, FNA Mapping‐based cytology can more accurately predict the presence of sperm on individual testis tissue specimens than can histopathology. One of the limitations of the FNA technique is that experienced cytologists, instead of histopathologists, are needed to accurately interpret and report the results, and expertise in these disciplines does not necessarily overlap.

Unlike with FNA Mapping in which information is obtained from many sites in the testis by design,^58^ during microdissection TESE, except when a biopsy specimen is taken during the procedure, the only information garnered about the process of spermatogenesis within the testis is live tissue examination for the presence or absence of sperm. There is no objective information learned about any potential variations in the histology pattern of the whole testis. Thus, FNA Mapping is far superior to microdissection TESE in terms of its informational and archival capacity regarding NOA testis biology. Such global geographical information is likely to be very valuable in the future to determine whether individuals with NOA could be candidates for stem cell and other cell‐based therapies.

### 3.5 Overview of each current testicular sperm retrieval technique

Historically, conventional TESE was introduced via publication in 1995,^59^ followed by Testicular Sperm Aspiration (TESA) in 1996.^60^ In OA and some NOA cases, these procedures are sufficient to retrieve testicular sperm for ICSI.^61^ The main advantages of these procedures are that they are simple and safe and can be done under local anesthesia.^62^ However, in more challenging NOA cases, these procedures can fail to find sperm for reasons outlined above, and more of the testis must be examined to increase the chance of retrieving sperm.^63^, ^64^, ^65^, ^66^, ^67^, ^68^

The first reported approach to finding sperm in more complex cases in which TESA/TESE procedures failed was systematic FNA biopsy, also termed FNA Mapping or Sperm Mapping. This technique demonstrated the complex heterogeneity of spermatogenesis in the testes in 1997.^11^ Inspired by the heterogeneity of spermatogenesis, microdissection TESE was developed and subsequently published in 1998.^14^ Japanese urologists were also fascinated by this procedure^69^, ^70^, ^71^ because, in part, it requires microsurgical skill, which was attractive to well‐trained reproductive urologists.

In cases of Klinefelter syndrome, the advantages of microdissection TESE are obvious.^72^, ^73^, ^74^ Because the testicular volume in Klinefelter syndrome is diminutive, finding sperm is comparatively easy with a surgical microscope. However, in cases of larger testes, as with most other NOA cases, it can be more difficult to find sperm even with a surgical microscope due to the increased volume of testicular tubules that need to be explored.^75^ Initially, microdissection TESE was performed concurrently with IVF egg retrieval.^26^ In cases where microdissection TESE failed, oocytes that were initially discarded, but with advances in egg freezing, including vitrification, they can now be frozen, eliminating unnecessary IVF cycles performed during failed sperm retrieval cases, and retrieved sperm are frozen and thawed for future use. However, it is still controversial whether using frozen–thawed testicular sperm results in equivalent ART outcomes compared to fresh sperm.^76^, ^77^ In most cases, especially when abundant numbers of sperm are found, there is no disadvantage to using frozen–thawed testicular sperm with IVF‐ICSI. But, in cases of harvesting (a) very few sperm or (b) sperm with no motility or (c) in poor female responders with few eggs, frozen–thawed sperm may be suboptimal when compared to fresh testicular sperm. This is primarily because although TESE sperm is largely immotile whether fresh or frozen–thawed, sperm viability (and therefore usability) approaches 90% when fresh and only 45% when thawed. An alternative approach now employed to reduce egg “wastage” in cases of failed sperm retrieval is to harvest and vitrify oocytes in advance of testicular sperm extraction and thaw eggs only if fresh sperm are found. This approach has met with favorable outcomes.^78^ While these issues are always a consideration with microdissection procedures, they are largely avoided with FNA Mapping and map‐directed TESE techniques as sperm retrieval procedures are uniformly successful (80%–100%) if the presence of sperm is known in advance. Through its ability to reliably allow fresh TESE sperm to be used with freshly retrieved eggs, FNA Mapping dramatically reduces the need to use frozen–thawed TESE sperm and the need to freeze uninjected oocytes.

There are other logistical disadvantages of microdissection TESE: it requires advanced surgical microscopy equipment, and proficiency in microsurgery. The testicular sperm retrieval rate depends on the skill and experience of the microsurgeon, and thus a high level of microsurgical skill is required.^79^ FNA Mapping is a procedure requiring only standard needles and a syringe holder, microscope slides and slide containers and is performed under local anesthesia. Expertise in cytological interpretation is critical for FNA Mapping, but since stained and fixed testis tissue slides can be shipped, this can and has been delegated to centers of excellence.

Interestingly, the sperm detection rate with FNA Mapping appears to have increased with the number of samples taken, a phenomenon not known to be true with microdissection TESE.^43^ It has been shown that when performing up to 18 aspiration FNA sites per testis, the ability to find testicular sperm reaches a plateau, and further sampling is unlikely to yield sperm.^44^ The false‐negative rate for FNA Mapping is currently unpublished but appears to be <5% in experienced hands. The low false‐negative rate with FNA Mapping is important, as NOA patients with negative diagnostic FNA Mapping can comfortably avoid unnecessary and more invasive testicular sperm extraction surgeries. The disadvantage of FNA Mapping is that it requires training of providers to be able to consistently and reliably retrieve quality cytology samples and to produce good cytologic smears.

What about the outcome in cases of patients with NOA azoospermia and clinical varicocele? It was reported that 2 out of 10 varicocele repairs in azoospermic patients resulted in pregnancies.^80^ A case was reported where, even after microdissection TESE failure, microsurgical varicocelectomy resulted in the appearance of ejaculated sperm.^81^ In azoospermic cases with varicocele, the decision to repair the varicocele before TESE/microdissection TESE is controversial,^82^, ^83^, ^84^, ^85^, ^86^, ^87^, ^88^, ^89^ although the majority of reports support the advantages of varicocele repair. Varicocele repair is reported to improve the histopathology of testes^90^; however, such cases are limited. The rate of ejaculated sperm appearance after varicocele repair is quite low. ART outcomes may improve after varicocele repair if TESE/microdissection TESE successfully retrieves testicular sperm.^91^, ^92^, ^93^ However, in cases where ejaculated sperm do not appear or TESE/microdissection TESE does not succeed in retrieving testicular sperm, varicocele repairs are deemed completely without merit, which is the outcome in the majority of cases.

If testicular sperm are found with diagnostic FNA Mapping, then varicocele repair could be more rationally recommended based on the cytologic findings to improve testicular sperm production. This could either (a) reduce the invasiveness of microdissection TESE procedures or (b) eliminate the need for invasive microdissection TESE procedures if ejaculated sperm appear after repair.

What do international societies think about the various approaches to testicular sperm retrieval in NOA cases? The European Association of Urology (EAU) Guidelines note that there is “low‐quality evidence” supporting the routine use of testicular fine‐needle mapping as an alternative diagnostic and predictive tool before TESE in men with NOA.^94^ The American Urological Association and American Society of Reproductive Medicine (AUA/ASRM) guidelines recommend microdissection TESE for NOA,^95^ but these recommendations are also based on low‐quality evidence. Meanwhile, the EAU guidelines comment that there is no distinct difference between conventional TESE and microdissection TESE for NOA.^94^ This discrepancy has led to dispute,^96^, ^97^ which has thus far been unproductive.

The JUA published its *Clinical Practice Guideline for Male Infertility* ^29^ and noted that microdissection TESE received a grade A recommendation despite having only level II evidence. In summary, the evidence used for societal recommendations on the best method of sperm detection and retrieval in NOA cases is based on low‐quality evidence and therefore weakens any generalizable recommendation, as well as making them subject to change with more data over time. This opens the possibility for considering viable alternatives to microdissection TESE, including FNA Mapping.

One issue of concern is that Japanese and other Asian males have been reported to have lower testicular sperm retrieval rates with microdissection TESE compared to rates reported in the USA and Europe.^98^ The reasons for this might be complex and could involve ethnic variations in disease. It could also reflect the fact that microsurgery training is not generally required to perform microdissection TESE procedures among Japanese providers, so the quality of microdissection TESE procedures could vary more widely. This observation is supported by the fact that repeat microdissection TESE procedures have found sperm when initial procedures have failed.^99^, ^100^ The lack of a “standard of care” in expertise for microdissection TESE procedures is in marked contrast to the way in which FNA Mapping was originally published with its inherent quality control mechanisms built into the procedure. FNA Mapping proposed a quality control model in which each aspiration sample is individually judged by defined cellular criteria as “adequate” or “inadequate” before being formally analyzed for sperm.^9^, ^10^ The idea behind this quality control concept is that samples with insufficient cells present do not reflect the true potential of that area of the testicle to harbor sperm. In addition, likely due to concerns about inducing hypogonadism, there are some reports which argue the validity of performing microdissection on the contralateral testis in cases where microdissection on the unilateral testis failed to retrieve testicular sperm.^101^, ^102^ However, with the far less invasive procedure, performing bilateral procedures is always recommended given the 19% published rate of side‐to‐side variation in sperm presence using this technique.

### 3.6 What Else can we Learn from FNA mapping and microdissection TESE?

In the era of testicular sperm‐ICSI, new terminology describing spermatogenesis throughout the entire NOA testes is needed beyond the current single‐site pathological classification. SCO cases with focal spermatogenesis could be termed SCO dominant. Because there are no strict criteria to differentiate between MA with and without sperm, MA cases with no mature sperm should be called uniform MA^8^ and MA cases with limited mature sperm production might be called dominant MA. In this respect, FNA Mapping is the only comprehensive method to demonstrate this global heterogeneity of spermatogenesis. In microdissection TESE, all the tissues without sperm except biopsy specimen are discarded, which makes it difficult to evaluate the heterogeneity of spermatogenesis throughout the entire testes in an objective way for reproductive purposes. FNA Mapping does not compete with, but rather is complementary to, microdissection TESE because both methods aim at the same purpose: higher sperm retrieval success with lower complication. Thus, all physicians could relearn the pathophysiology of azoospermia through FNA Mapping and microdissection TESE.

## 4 CONCLUSION

In the absence of randomized trial data and according to the best (Cochrane) data to date, there is no definitive “gold standard” procedure for testicular sperm retrieval in NOA cases.^94^, ^95^ In this setting, microdissection TESE and FNA Mapping followed by directed TESE procedures are both viable approaches to finding sperm in NOA men. The advantages and disadvantages of these two approaches to testicular sperm retrieval were reviewed here. Although microdissection TESE is a single‐step procedure for finding and retrieving testicular sperm, FNA Mapping is a two‐step method to predict the success of retrieving testicular sperm in advance of an intended sperm retrieval procedure. Despite demonstrating similar overall sperm retrieval rates, these two approaches differ widely in (a) their level of invasiveness, (b) their degree of induced hypogonadism, (c) their ability to predict successful sperm retrieval, and (d) their reliability in avoiding unnecessary sperm retrieval procedures. A large advantage of FNA Mapping in NOA patients at higher risk for hypogonadism is that it is the only method that predicts testicular sperm retrieval in advance and also guides physicians in choosing potentially less invasive procedures (i.e. TESA, TESE) for retrieving testicular sperm. We propose that, instead of recommending either microdissection TESE or FNA Mapping to all patients, clinicians should thoughtfully determine which procedure is best for each individual patient. This also requires that physicians be trained and experienced in both techniques, which will improve patient care and significantly enhance outcomes in the field of andrology.

## CONFLICT OF INTEREST STATEMENT

The authors declare no conflicts of interest for this article.
